# Understanding Clinician Knowledge About Race Adjustment in the Vaginal Birth After Cesarean Calculator

**DOI:** 10.1089/heq.2023.0049

**Published:** 2024-01-08

**Authors:** Julia Cron, Amelia A. Shapiro, Laura Carasimu, Julia Iyasere, Johanna M. Schisler, Szilvia Nagy, Sandra Angus, Anna Burgansky, Ashlesha K. Dayal, Tracy Bohn Hemmerdinger, Denise Howard, Corrina Oxford-Horrey, Donald C. Phillibert, Jean-Ju Sheen, Dena Goffman

**Affiliations:** ^1^ Department of Obstetrics and Gynecology, Weill Cornell Medicine, New York, New York, USA.; ^2^ Dalio Center for Health Justice, NewYork-Presbyterian, New York, New York, USA.; ^3^ Women's Service Line, NewYork-Presbyterian, New York, New York, USA.; ^4^ Department of Obstetrics and Gynecology, Columbia University Irving Medical Center, New York, New York, USA.; ^5^ Department of Quality and Patient Safety NewYork-Presbyterian, New York, New York, USA.

**Keywords:** obstetrics, racial minority, health disparities, clinical algorithm, race

## Abstract

Disparities in maternal health outcomes are striking. Historical and biased clinical support tools have potential to exacerbate inequities. In 2022, NewYork-Presbyterian, with ∼25,000 annual births, and our academic partners, Columbia and Weill Cornell, launched a program to better understand practice patterns and clinician attitudes toward a vaginal birth after cesarean (VBAC) calculator, which predicts VBAC success. This article summarizes the program, focusing on the VBAC calculator utilization survey, which measured provider awareness of the revised calculator and key factors considered in patient counseling. Our preliminary findings warrant future research and education on the calculator's implications for counseling and outcomes.

## Introduction

In 2007, the National Institute of Child Health and Human Development (NICHD) published a vaginal birth after cesarean (VBAC) success calculator, which relied on age, body mass index, history of vaginal delivery, indication for prior cesarean, history of VBAC, and race and ethnicity as model inputs.^1^ Vyas et al.^2^ warned of the danger of including race within a clinical algorithm as it implicitly accepts historical and social constructs as biological. Rather, race is a proxy for variables that demonstrate the impact of racism on health.

Adaptation of the 2007 calculator outside of the United States, which excluded race and ethnicity, demonstrated the insignificance of race and ethnicity and noted improved performance of the model with exclusion of ethnicity.^3–5^ Subsequently, in 2021, the American College of Obstetricians and Gynecologists acknowledged the inappropriateness of including race and ethnicity in such a model and issued a practice advisory highlighting the limitations of the VBAC calculator and advising clinicians about the availability of a revised calculator without race and ethnicity.

Given the increasing recognition that differences in outcome by race are not biologically based but rather reflect the impact of systemic racism, social determinants of health, and clinician bias, utilizing race and ethnicity variables in a VBAC calculator may deter patients and clinicians from [Trial of Labor After Cesarean] (TOLAC) without biologic cause and thereby reinforce inequity rather than support patient-centered care.^6^

Since then, a recent study of a racially diverse population at an urban tertiary medical center demonstrated that the 2021 calculator more accurately predicted VBAC success and the 2007 calculator underestimated success rates for Black and Hispanic patients.^7^ The authors note that the revised calculator has the potential to impact shared decision making, encourage more patients to undergo trial of labor after cesarean (TOLAC), and reduce the racial and ethnic disparities in maternal morbidity.

In 2021, the New York City (NYC) Department of Health and Mental Hygiene announced the formation of the Coalition to End Racism in Clinical Algorithms (CERCA) following the Board of Health's landmark resolution declaring racism a public health crisis. NewYork-Presbyterian (NYP) joined 10 other health systems in CERCA's 1st year, to create a community of support for health systems working to eliminate bias and race correction in clinical algorithms.

Health care providers increasingly use predictive models and algorithms to guide treatment decisions; however, these tools can bring unintended bias, which can lead to inequitable outcomes across groups.^8^ Both through CERCA and in other operations, NYP commits to analyzing algorithms and predictive models to assess for bias and unfairness, and taking action when either is identified.

NYP is an academic health system, affiliated with Columbia University Vagelos College of Physicians and Surgeons and Weill Cornell Medical College, based in NYC and Westchester County, with obstetric services at eight hospital campuses, delivering ∼25,000 babies per year. Our commitment to excellence in medical education, groundbreaking research, and innovative patient-centered clinical care includes a profound commitment to equity and health justice, with a substantial body of work in addressing inequities in maternal morbidity and mortality.

In our 1st year in CERCA, NYP focused on an assessment of clinical decision support tools to predict VBAC success. Although the NICHD VBAC calculator has not included race since December 2021,^9^ we recognized that additional study and education on this topic are warranted. In 2022, NYP launched a provider survey to better understand practice patterns and clinician knowledge about the VBAC calculator. The primary goal of the survey was to measure provider awareness of the 2021 elimination of race-based corrections in the NICHD VBAC algorithm and understand provider perceptions of VBAC calculator components. This information would help inform future faculty and staff training and education, as well as review clinical decision-making tools in use throughout our health system.

## Methods

An 11-item Qualtrics survey (Table 1) was developed by NYP's Race Correction in VBAC workgroup, with input from NYP's obstetrical leadership, department of quality and patient safety, and Dalio Center for Health Justice. The survey was administered to 356 obstetrics and gynecology attendings, residents, fellows, physician assistants, nurse practitioners, and midwives across eight NYP campuses offering obstetrical services. The survey was distributed by email through physician leadership, and remained active for 8 weeks with periodic reminders to recipients.

**Table 1.** Survey Questions—Understanding Clinician Attitudes Toward Race Adjustment in the VBAC Calculator

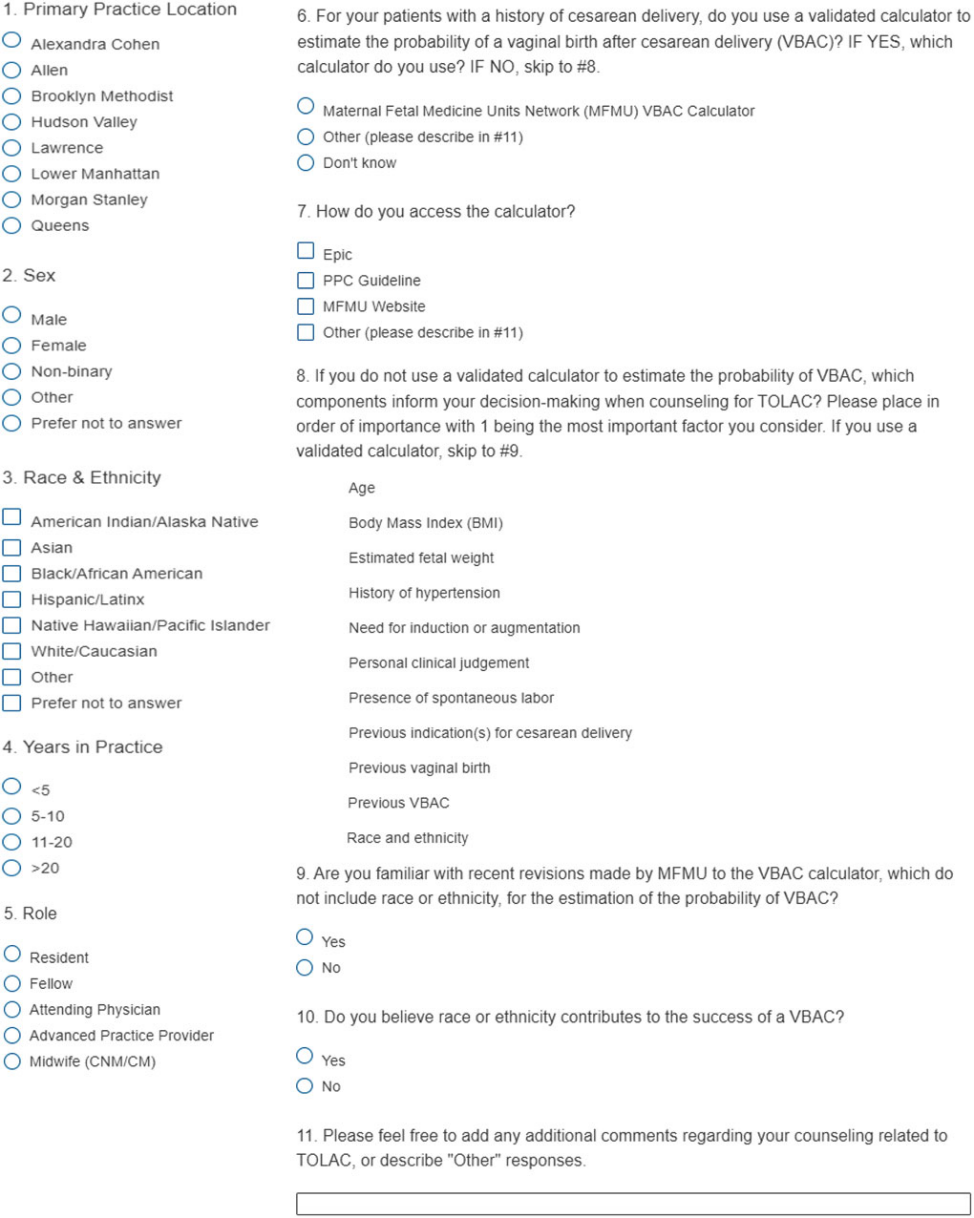

CM, Certified Midwife; CNM, Certified Nurse Midwife; PPC, Perinatal Practice Council; TOLAC, trial of labor after cesarean; VBAC, vaginal birth after cesarean.

Questions were developed to gauge provider demographics (location, years in practice, provider type, race/ethnicity, and gender). Respondents were asked whether they use a validated VBAC calculator, and if so, how they access it. Respondents who did not report using a VBAC calculator were asked to rank-order 11 factors (Fig. 1), derived from a focus group centered around VBAC counseling, that might inform their decision making.

## Results

After 8 weeks of survey collection, 125 survey responses were collected and aggregated to clarify clinician attitudes toward the VBAC calculator. Key demographics are summarized in Table 2.

**Table 2.** Survey Respondent Demographics

	% of total surveys
Provider type	
Attending physicians	74
Residents and fellows	18
Advanced practice providers (PA, NP, midwife)	8
Experience	
% over 20 years	27
% 5–20 years	47
% under 5 years	27

NP, Nurse Practitioner; PA, Physician Assistant.

More than half (65%) of providers responded that they use a validated calculator to estimate the probability of a successful VBAC. Twenty-three percent of responding providers were unaware of the 2021 revisions to the VBAC calculator that removed race and ethnicity from the algorithm, and 10% reported believing that race or ethnicity contribute to the success of a VBAC.

Of respondents who do not use a validated calculator, previous vaginal birth, previous indications, and previous VBAC were the top-ranked factors among respondents, with 57% including all three of these factors in their top three, and 77% including at least one of these factors in their top three. All respondents ranked race and ethnicity in the bottom three factors for consideration, and 83% ranked race and ethnicity in last place for consideration.

## Discussion

Developing and deploying the survey of current practices for VBAC calculator utilization was one early effort in NYP's larger focus around maternal health equity. The survey provided baseline information about provider awareness and knowledge about the VBAC calculator. We acknowledge the limitations of the calculator and recognize the many factors that influence counseling around VBAC, including provider biases, birthing location, and historical context.

Nevertheless, we believe the revised VBAC calculator has the potential for improving racial equity as it provides an objective measure with which to begin patient-centered shared decision-making conversations, and without a defined “cutoff” for success, it could serve as a “springboard” with which to begin the nuanced important discussion about risks and benefits of TOLAC.

Our survey highlighted that there are still gaps in knowledge about the revised calculator as well as underutilization. As an output from the survey work, NYP's Race Correction in VBAC workgroup presented CERCA's mission of removing race adjustment from clinical algorithms, as well as survey background and findings to the Perinatal Practice Council (PPC)—a 90-member system-wide multidisciplinary committee dedicated to establishing standards in clinical practice and broadcasting communications to clinicians within the heath system.

The workgroup also developed a short educational piece about race correction in the VBAC calculator, which was shared with the PPC, along with related articles and peer-reviewed literature. PPC members will be disseminating the learning to cross-campus teams in a variety of ways, including through interdisciplinary tiered huddles, which keep teams apprised of key updates. Furthermore, NYP contracted with a national vendor for a perinatal-specific implicit bias training course. The new curriculum will be implemented this year and will help to address the visible and invisible ways that both conscious and unconscious racial stereotypes affect the quality and equity of patient care.

Importantly, 23% of our clinicians were not aware of the new calculator and 10% did believe that race plays a role. Thus, we now have evidence that education is imperative. After our interventions, a postsurvey will be deployed to gather feedback from the training sessions, and reassess provider attitudes and practices.

NYP will continue to focus on supporting patient-centered shared decision-making models. Tools such as the Birth Preferences form, Respectful Care at Birth brochure, and Patient Reported Experience Measure survey inform, educate, and support patients, and aid in developing trusting relationships with their care team. NYP's Birth Equity Committee is foundational to identifying how individual and systemic racism impacts birth outcomes, and taking action to improve both the experience of care and perinatal outcomes for Black birthing people in the communities we serve. The standards set by this committee help guide our teams to respect and be aware of patients' human rights during pregnancy, labor, and childbirth.

## Abbreviations Used

CERCA: Coalition to End Racism in Clinical Algorithms
NICHD: National Institute of Child Health and Human Development
NYC: New York City
NYP: NewYork-Presbyterian
PPC: Perinatal Practice Council
TOLAC: trial of labor after cesarean
VBAC: vaginal birth after cesarean
